# Belun Sleep Platform versus in-lab polysomnography for obstructive sleep apnea diagnosis

**DOI:** 10.1007/s11325-025-03433-w

**Published:** 2025-08-07

**Authors:** Vipada Tirachaimongkol, Wish Banhiran, Wattanachai Chotinaiwattarakul, Sarin Rungmanee, Chawanon Pimolsri, Jindapa Srikajon, Navarat Kasemsuk

**Affiliations:** 1 Department of Otorhinolaryngology, Faculty of Medicine Siriraj Hospital, Mahidol University, 2 Wanglang Road, Bangkok Noi, Bangkok, 10700 Thailand; 2 Siriraj Sleep Center, Faculty of Medicine Siriraj Hospital, Mahidol University, Bangkok, Thailand

**Keywords:** Belun Sleep Platform, Home sleep apnea testing, Obstructive sleep apnea, Polysomnography

## Abstract

**Objective:**

We aimed to compare the Belun Sleep Platform (BSP), an artificial intelligence-driven home sleep testing device, with polysomnography (PSG) for diagnosing obstructive sleep apnea. The BSP analyzes oxygen saturation, heart rate, and accelerometry patterns.

**Methods:**

Participants scheduled for PSG and with no significant cardiovascular or neuromuscular comorbidities were recruited. They underwent simultaneous in-laboratory, full-night PSG with the BSP. We assessed diagnostic properties, including sensitivity, specificity, positive predictive value, negative predictive value, and accuracy.

**Results:**

A total of 40 participants (54.3% male) with a mean age of 49.9 years were enrolled. For an apnea-hypopnea index (AHI) cutoff of ≥ 15 events/h, BSP showed an accuracy of 68.5%, sensitivity of 35.2%, and specificity of 100% under American Academy of Sleep Medicine criteria 1 A and 1B. For AHI thresholds of ≥ 5 and ≥ 30 events/h, sensitivity was 82.1% and 33.3%, respectively, while specificity was 14.2% and 100%, respectively. BSP-AHI correlated moderately with PSG-AHI (intraclass correlation coefficient [ICC] = 0.737). BSP’s oxygen desaturation index (ODI) showed a strong correlation with PSG-ODI (ICC = 0.882). Moderate correlations were observed between BSP and PSG for non-rapid eye movement sleep duration (ICC = 0.736), rapid eye movement sleep duration (ICC = 0.664), total sleep time (ICC = 0.617), and sleep efficiency (ICC = 0.719).

**Conclusions:**

The BSP’s high specificity but low sensitivity suggests it serves better as a confirmatory tool rather than a primary screening method. Its moderate concordance with PSG underscores its potential in settings where PSG is unavailable. However, further investigation is needed to refine its clinical applications.

## Introduction

Obstructive sleep apnea (OSA) involves intermittent partial or complete obstruction of the upper airway during sleep. It is associated with multiple adverse health outcomes, including cardiovascular, metabolic, and cerebrovascular morbidities, as well as neurocognitive impairment and mortality [1–7]. The prevalence of OSA in the general adult population, defined by an apnea-hypopnea index (AHI) of five or more events per hour, ranges from 9 to 38%. Rising obesity rates and aging populations may further increase its burden [1–3]. 

Polysomnography (PSG) remains the gold standard for diagnosing OSA, but it has notable drawbacks. These include high costs, long wait times, and the need for skilled technologists and specialists, especially in resource-limited settings. Consequently, alternative sleep tests have garnered attention. The American Academy of Sleep Medicine (AASM) advises using a technically adequate home sleep apnea testing device for diagnosing OSA in uncomplicated, high-risk adults [8, 9]. Advanced versions of these devices that incorporate pulse oximetry, photoplethysmography, accelerometry, and heart rate variability have recently emerged.

The US Food and Drug Administration-approved Belun Sleep Platform (BSP) is a new home sleep apnea testing device worn on the proximal index finger of the non-dominant hand. It uses sensors for pulse oximetry, photoplethysmography, and a three-axis accelerometer to measure signals from the radialis indicis artery. An artificial intelligence algorithm processes SpO₂, heart rate, heart rate variability, accelerometry, and photoplethysmography waveforms to detect respiratory events and estimate total sleep time in five-minute segments [10]. The BSP demonstrated a sensitivity of 0.85 and a specificity of 0.87 for classifying AHI ≥ 15 events per hour [11]. However, few studies have evaluated its diagnostic performance, and only a limited number of full-night assessments exist for comparison.

This study aimed to compare the BSP’s diagnostic characteristics—sensitivity, specificity, positive predictive value (PPV), negative predictive value (NPV), and accuracy—with those of full-night PSG for diagnosing OSA.

## Materials and methods

### Study design and ethical approval

This cross-sectional diagnostic accuracy study took place at the Department of Otorhinolaryngology, Faculty of Medicine, Siriraj Hospital, Thailand. It was approved by the Siriraj Institutional Review Board (reference: Si-177/2024) and was reported according to the STARD 2015 guidelines for diagnostic accuracy studies.

### Participants

Adults older than 18 years who were scheduled for a full-night PSG at Siriraj Hospital, Mahidol University, were screened for eligibility and enrolled. The exclusion criteria were the use of oxygen or noninvasive ventilation, as well as a history of heart failure, atrial fibrillation, chronic obstructive pulmonary disease, neuromuscular disorders, insomnia, parasomnia, unstable cardiopulmonary status, or hospitalization within the preceding 30 days. Informed consent was obtained from all participants. Each participant underwent full-night PSG (the reference standard) while simultaneously wearing the BSP as the index test.

### Sample size calculation

The sample size was determined using the nQuery Adviser Program based on the confidence interval for intraclass correlation coefficient (ICC) and the K measurement formula. An ICC of 0.75, indicating moderate to good reliability, was chosen in accordance with guidelines from *A Guideline of Selecting and Reporting Intraclass Correlation Coefficients for Reliability Research* [12]. A 95% confidence interval of 0.15 and a reference ICC value of 0.882 from a study by Ou et al. [13] were applied. To accommodate potential dropouts, the final target was increased by 20%, yielding a sample size requirement of 40 participants.

### BSP

**Fig. 1:**
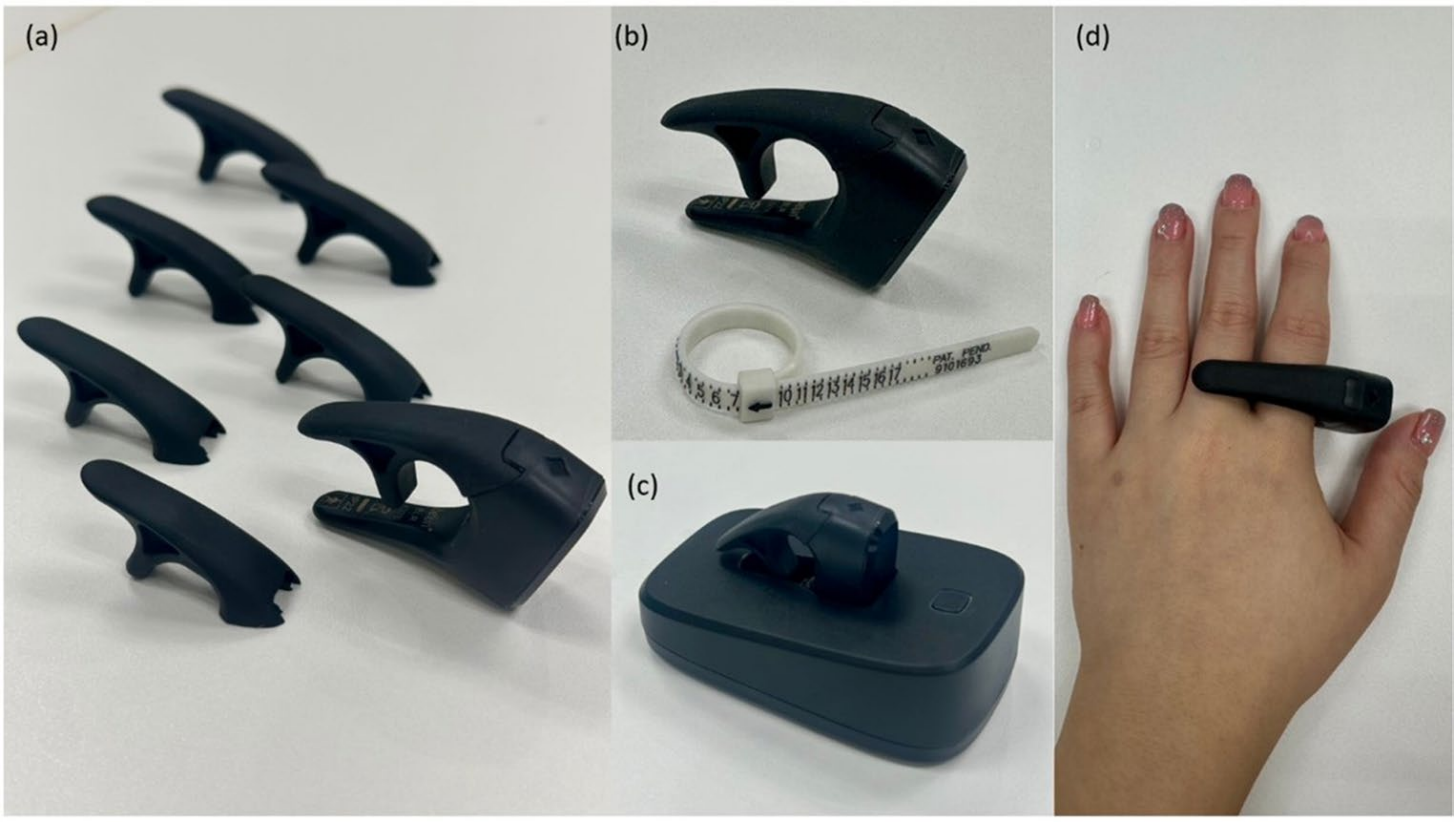
Belun Sleep Platform (BSP) home sleep apnea testing device components.(**a**) Ring pulse oximeters in multiple sizes; (**b**) paper ring selector for measurement; (**c**)

The BSP consists of three primary components: a ring pulse oximeter, a charging cradle, and cloud-based software. The ring pulse oximeter (Belun Technology, Hong Kong) is integrated into the wearable device. It utilizes reflectance photoplethysmography sensors located on the inner side of the ring to measure oxygen saturation (SpO₂), heart rate, and waveform data from the radialis indicis artery. The device is US FDA-cleared and CE-marked. The BSP is available in eight-ring sizes (5 to 12), corresponding to circumferences of 15.7 mm to 22.2 mm. Figure 1 shows the BSP devices and a paper ring selector for sizing.

A sleep technician measured the proximal phalanx of the index finger of each participant’s non-dominant hand using the paper ring selector. The ring was then secured for overnight monitoring. After each sleep test, the device was placed in its charging cradle, and the recorded data were uploaded to the BSP cloud. A proprietary artificial intelligence algorithm automatically processed these data, generating a report for immediate access. The study team remained blinded to the PSG results while interpreting BSP data.

### Full-night PSG

A full-night in-lab PSG was performed according to a standardized protocol, with recordings manually scored in 30-second epochs based on the AASM Scoring Manual, version 3. An obstructive apnea was defined as a reduction in thermistor airflow to less than 10% of baseline for more than 10 s, with continued respiratory effort. A hypopnea event was defined as a ≥ 30% reduction in nasal pressure signal excursions for more than 10 s, accompanied by either ≥ 3% oxygen desaturation and/or arousal (criterion 1 A) or ≥ 4% oxygen desaturation (criterion 1B). Scoring technicians and certified sleep physicians remained blinded to BSP results to prevent bias.

### Statistical analysis

Categorical variables were expressed as counts and percentages, whereas continuous variables were reported as mean with standard deviation for normally distributed data, or median (IQR) for non-normally distributed data. Group comparisons for continuous data employed unpaired *t*-tests (normally distributed) or Mann‒Whitney *U* tests (non-normally distributed). Intraclass correlation assessed the reliability of the index test. Diagnostic properties—sensitivity, specificity, PPV, NPV, positive likelihood ratio, negative likelihood ratio, and accuracy—were evaluated using two-by-two tables. The area under the receiver operating characteristic curve (AUROC) was also calculated. The correlations were evaluated between ODI from BSP and PSG (both 3% and 4%), as well as between AHI, total sleep time, sleep efficiency, REM and NREM durations, and minimum oxygen saturation derived from both BSP and PSG. All analyses were conducted using IBM SPSS Statistics, version 27 (IBM Corp, Armonk, NY, USA). A two-sided *P* value below 0.05 was considered statistically significant.

## Results

### Study population

A total of 40 participants were initially enrolled in the study. Five patients were excluded due to incomplete BSP data, leaving 35 participants for final analysis. Among these, 19 were male (54.3%), with an average age of 49.9 ± 15.9 years and a mean body mass index of 25.6 ± 5.4 kg/m². Of the 35 participants, 24 (22%) were classified as high risk based on a STOP-Bang score of ≥ 3, and 12 (34.3%) had comorbid hypertension. PSG recorded an average baseline AHI of 21.5 ± 19.1 events/h under AASM criterion 1 A and 19.6 ± 17.4 events/h under criterion 1B. Additional baseline characteristics are presented in Table 1.

**Table 1:** Baseline characteristics of the 35 enrolled participants

Demographic data	Results (*n* = 35)
Male*†*	19 (54.3)
Age, years*‡*	49.9 ± 15.9
STOP-Bang ≥ 3*†*	24 (68.6)
Comorbid hypertension*†*	12 (34.3)
• HT on beta-blocker	4 (11.4)
• HT on CCB	6 (17.1)
*PSG results: AASM criterion 1 A†*	
• AHI < 5	7 (20)
• AHI 5–14.9	11 (31.4)
• AHI 15–29.9	8 (22.9)
• AHI ≥ 30	9 (25.7)
*PSG results: AASM criterion 1B†*	
• AHI < 5	8 (22.9)
• AHI 5–14.9	10 (28.6)
• AHI 15–29.9	10 (28.6)
• AHI ≥ 30	7 (20)
*Other PSG parameters‡*	
• TRT, minutes	486.3 ± 75.9
• TST, minutes	388.0 ± 27.3
• SE, %	79.4 ± 14.8
• Minimum SpO2, %	84.6 ± 8.9
• 3% ODI, events/hour	12.6 ± 16.0
• T90, %	1.6 ± 6.5

Abbreviations: AASM, American Academy of Sleep Medicine; AHI, apnea-hypopnea index; BMI, body mass index; CCB, calcium channel blocker; HT, hypertension; PSG, polysomnography; TRT, total recording time; TST, total sleep time; SE, sleep efficiency; ODI, oxygen desaturation index; T90, percentage of sleep time with SpO₂ below 90% † Values are presented as number (percentage); ‡ as mean ± SD

### Diagnostic properties

Table 2 provides detailed information on the diagnostic properties of BSP compared to PSG, as well as the properties of the combined BSP with STOP-Bang ≥ 3, according to both AASM criteria. These properties include sensitivity, specificity, PPV, NPV, and accuracy.

**Table 2:** Diagnostic performance of the Belun Sleep Platform (BSP) compared with full-night polysomnography (PSG), and combined BSP with STOP-Bang ≥ 3, based on AASM scoring criteria

Cutoff value	Accuracy	Sensitivity	Specificity	PPV	NPV	LR+	LR–	AUROC
*BSP to PSG according to AASM criteria 1 A *(*n = 35)*								
AHI ≥ 5	68.57 (50.71–83.15)	82.14 (63.10–93.94)	14.29 (0.36–57.87)	79.31	16.67	0.96	1.25	0.48
AHI ≥ 15	68.57 (50.71–83.15)	35.29 (14.21–61.67)	100.00 (81.47–100)	100.00	62.07	NA	0.65	0.67
AHI ≥ 30	82.86 (66.35–93.44)	33.33 (7.49–70.07)	100.00 (86.77–100)	100.00	81.25	NA	0.67	0.67
*BSP to PSG according to AASM criteria 1B (n = 35)*								
AHI ≥ 5	71.43 (53.70–85.37)	85.19 (66.67–95.81)	25.00 (3.19–65.09)	79.31	33.33	1.14	0.59	0.55
AHI ≥ 15	68.57 (50.71–83.15)	35.29 (14.21–61.67)	100.00 (81.47–100.00)	100.00	62.07	NA	0.65	0.68
AHI ≥ 30	88.57 (73.26–96.80)	42.86 (9.90–81.60)	100.00 (87.66–100.00)	100.00	87.50	NA	0.57	0.71
*Combined STOP-Bang with BSP to PSG according to AASM criteria 1 A (n = 24)*								
AHI ≥ 5	75.00 (53.29–90.23)	90.00 (68.30–98.77)	0.00 (0.00–60.00)	81.82	0.00	0.90	NA	0.45
AHI ≥ 15	66.67 (44.68–84.37)	38.46 (13.86–68.42)	100.00 (71.50–100.00)	100.00	57.89	NA	0.61	0.69
AHI ≥ 30	79.17 (57.85–92.87)	37.50 (8.52–75.51)	100.00 (79.40–100.00)	100.00	76.19	NA	0.62	0.68
*Combined STOP-Bang with BSP to PSG according to AASM criteria 1B (n = 24)*								
AHI ≥ 5	79.17 (57.84–92.87)	94.74 (73.97–99.88)	20.00 (0.50–71.64)	81.82	50.00	1.18	0.26	0.57
AHI ≥ 15	66.67 (44.68–84.37)	38.46 (13.86–68.42)	100.00 (71.50–100.00)	100.00	57.89	NA	0.61	0.69
AHI ≥ 30	83.33 (62.62–95.26)	42.86 (9.90–81.60)	100.00 (80.50–100.00)	100.00	80.95	NA	0.57	0.71

Abbreviations: AASM, American Academy of Sleep Medicine; AHI, apnea-hypopnea index; AUROC, area under the receiver operating characteristic curve; BSP, Belun Sleep Platform; LR–, negative likelihood ratio; LR+, positive likelihood ratio; NPV, negative predictive value; PPV, positive predictive value; PSG, polysomnography

### Correlation between BSP and PSG parameters

The BSP’s oxygen desaturation index (BSP-ODI) displayed a strong correlation with PSG-3%ODI (ICC = 0.882) and PSG-4%ODI (ICC = 0.898). BSP-AHI demonstrated a moderate correlation with PSG-AHI under criterion 1 A (ICC = 0.737) but showed a good correlation under criterion 1B (ICC = 0.786).

Other sleep metrics exhibited moderate correlations, including non-rapid eye movement sleep duration (ICC = 0.736), rapid eye movement sleep duration (ICC = 0.664), total sleep time (ICC = 0.617), sleep efficiency (ICC = 0.719), and minimum oxygen saturation (ICC = 0.696). In contrast, total recording time and the percentage of time spent with oxygen saturation ≥ 90% demonstrated poor correlations, with ICC values of 0.113 and 0.422, respectively.

### Bland-Altman plot

**Fig. 2:**
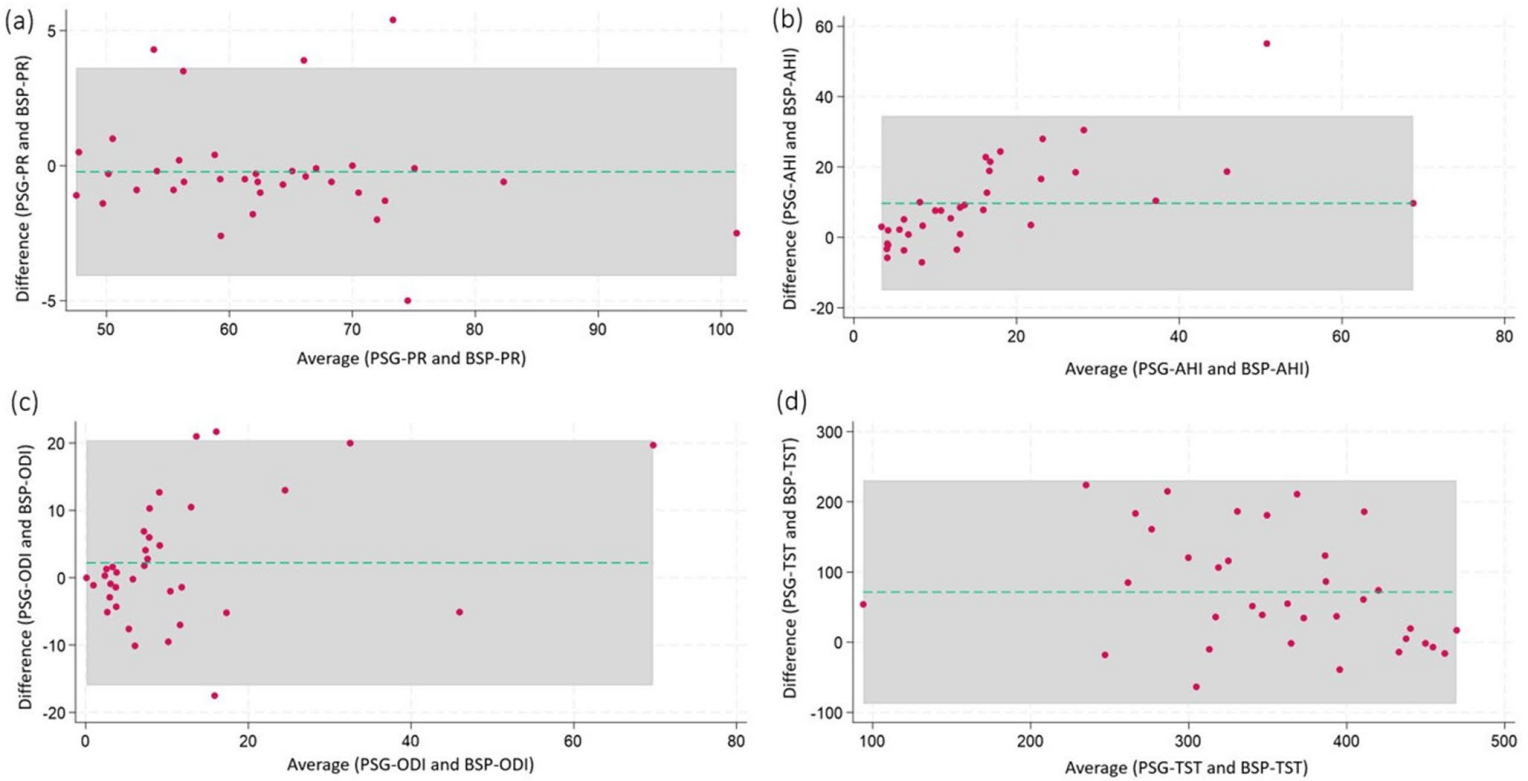
Bland-Altman plots demonstrating correlations between BSP and PSG parameters. (**a**) BSP-PR vs. PSG-PR; (**b**) BSP-AHI vs. PSG-AHI (AASM criterion 1 A)

A Bland–Altman analysis was performed to assess agreement between pulse rate (PR) measurements; the mean difference between the two methods was 0.23 bpm, with an overall correlation of 0.98. Figure 2 demonstrates a Bland–Altman plot for PR. AHI, ODI, and TST from BSP and PSG.

### Scatterplot observations

**Fig. 3:**
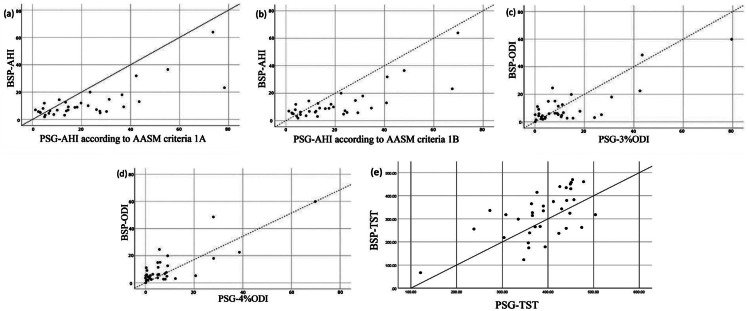
Scatterplots demonstrating correlations between BSP and PSG parameters. (**a**) BSP-AHI vs. PSG-AHI (AASM criterion 1 A); (**b**) BSP-AHI vs. PSG-AHI (AASM)

Figure 3 illustrates scatterplots comparing BSP-AHI and PSG-AHI under both AASM criteria, as well as BSP-ODI and PSG-ODI values. A moderate positive correlation was observed between BSP-AHI and PSG-AHI, although the relationship flattened at higher PSG-AHI values. Correspondingly, BSP-ODI and PSG-ODI exhibited a positive correlation. In both comparisons, data points trended below the 45-degree reference line at higher AHI and ODI ranges.

### Total respiratory event comparison

Utilizing the available AHI and TST data, the total number of respiratory events for both PSG and BSP was approximated by multiplying AHI by the total sleep time. The estimated mean total event count was approximately 139 events for PSG and 110 events for BSP. Despite this, the two measures showed a very strong positive correlation (*r* = 0.965, *p* < 0.001).

## Discussion

Several validation studies have been conducted on various home sleep apnea testing devices, including the BSP, yet only a limited number have directly compared BSP with full-night PSG for diagnosing OSA in adults. In this study, using an AHI threshold of ≥ 15 events/h, BSP demonstrated very high specificity under AASM scoring criteria 1 A and 1B. Its moderate accuracy (66.7%‒88.6%) aligned with the AUROC value, indicating reliability in detecting OSA in selected individuals. However, while BSP’s sensitivity was acceptable among patients with mild OSA, it remained low for moderate to severe OSA (AHI ≥ 15 events/h), suggesting that BSP may underestimate the true AHI in more severe cases. These findings point to BSP’s potential role as a confirmatory tool rather than as a sole screening method, particularly in resource-limited settings where PSG is unavailable.

In comparison to a previous study by Wenbo et al., [11] which reported high specificity for BSP, our study also found excellent specificity but documented lower sensitivity at the AHI ≥ 15 events/h cutoff. These differences may result from varying patient demographics, as Wenbo et al. enrolled a broader range of disease severity, and from methodological factors such as full-night in-lab PSG versus split-night or home testing. Similar discrepancies have emerged with other photoplethysmography and peripheral arterial tone-based devices, including WatchPAT and NightOwl [10, 11, 14–16]. 

The ICC revealed a high level of reliability between BSP-AHI and PSG-AHI for both AASM criteria, as well as between BSP-ODI and PSG-ODI. Nevertheless, BSP tended to overestimate AHI in those with AHI below 15 events/h and underestimate it in those with AHI of 15 or higher. Overestimation in milder cases may arise from the BSP algorithm’s detection of respiratory effort–related arousals or autonomic arousals unrelated to OSA, whereas underestimation in higher ranges may be due to difficulty detecting consecutive events in quick succession, excluding poor signals in very severe OSA, or a lack of specialized training to recognize heart rate variability patterns in central sleep apnea.

Because BSP has high specificity but low sensitivity, it appears best suited as a confirmatory rather than a primary screening device. Tests with high specificity effectively rule in a condition, so a positive BSP result strongly suggests OSA. However, the limited sensitivity prevents BSP from detecting many true OSA cases, diminishing its usefulness as a standalone diagnostic tool. Clinical practice might therefore benefit from combining BSP with high-sensitivity screening measures to reduce missed diagnoses.

The STOP-Bang questionnaire offers high sensitivity and moderate specificity for OSA detection [17]. Because BSP shows excellent specificity but limited sensitivity, we tested the combination of BSP with a STOP-Bang score of ≥ 3 to enhance sensitivity while retaining high specificity. Although this approach increased sensitivity to 0.38 at an AHI threshold of ≥ 15 events/h and maintained high specificity, overall accuracy remained at 66.6%, suggesting that the combined method is still inadequate for fully reliable OSA detection.

Strong correlations between BSP-ODI and PSG-ODI (at both 3% and 4% definitions) highlight BSP’s reliability in measuring oxygen desaturation events. Moderate correlations in non-rapid eye movement and rapid eye movement sleep duration, total sleep time, sleep efficiency, and minimum oxygen saturation indicate that BSP can approximate key facets of sleep architecture. However, poor correlation in total recording time and time spent with oxygen saturation ≥ 90% underscores the need for continued refinement to improve BSP’s precision in capturing particular sleep parameters.

The low correlation in TRT between BSP and PSG was noted in terms of other sleep parameters, which might be the result of variations in the definition and computation of recording time between the two systems. For example, PSG manually specifies TRT from “lights off” to “lights on” based on technician input and behavioral cues, while the BSP algorithm probably starts and stops recording depending on device wear detection and signal acquisition thresholds. TRT measurement inconsistencies may be caused by these methodological variations.

Bland–Altman plots demonstrated strong agreement between BSP and PSG for pulse rate, with minimal bias and narrow limits of agreement. For AHI and ODI, BSP tended to underestimate values at higher severities, with wider variability observed in lower ODI ranges. TST showed the greatest discrepancy, likely due to differences in sleep period detection algorithms. These findings support BSP’s accuracy for PR and ODI but highlight limitations in estimating AHI and TST.

In addition to individual comparisons of AHI and TST, the correlation between total respiratory event counts derived from both methods was also assessed. By multiplying AHI by total sleep time, the overall event burden was estimated, and a very strong correlation between BSP and PSG was observed. This finding suggests that, although BSP tends to underestimate AHI and TST individually, it still provides a consistent approximation of the total number of respiratory events. This strengthens BSP’s potential utility as a confirmatory diagnostic tool, particularly in settings where estimating the overall burden of disease is clinically relevant.

This study has limitations. First, it was conducted in a sleep lab with professional sleep technicians guiding device use, so the results may not fully represent BSP performance in real-world home conditions without staff assistance. Second, a small number of participants were taking heart rate–modifying medications, which might affect BSP’s performance. Also, some participants with severe OSA automatically transitioned to a split-night protocol, reducing the number of more severe cases. Our recruitment strategy primarily targeted higher-risk individuals, which may generate performance estimates that differ from those found in a general population. Finally, the BSP-generated report does not provide separate counts for specific respiratory event types, such as obstructive apneas and hypopneas. As a result, we were unable to perform the intended analysis based on distinct respiratory event classifications.

Future research should include larger sample sizes and broader patient populations, such as those with comorbidities, individuals on heart rate–modifying medications, more severe OSA cases, and people with low STOP-Bang scores. Such studies could clarify BSP’s role as an alternative diagnostic option for OSA in high-risk populations.

### Conclusions

BSP demonstrated high specificity and reliable oxygen desaturation detection but displayed limited sensitivity as a standalone OSA screening tool, especially for moderate to severe cases (AHI ≥ 15). It showed strong agreement with PSG for ODI measurements and moderate correlations for key sleep parameters but tended to overestimate mild OSA and underestimate more severe OSA. Given the controlled setting and selective study population, further investigation in real-world home environments with larger, more diverse samples is needed to refine BSP’s clinical utility.

## Data Availability

The data that support the findings of this study are not openly available due to reasons of sensitivity and are available from the corresponding author upon reasonable request.
